# *N*- and *O*-Glycosylation Pathways in the Microalgae Polyphyletic Group

**DOI:** 10.3389/fpls.2020.609993

**Published:** 2020-12-17

**Authors:** Elodie Mathieu-Rivet, Narimane Mati-Baouche, Marie-Laure Walet-Balieu, Patrice Lerouge, Muriel Bardor

**Affiliations:** ^1^UNIROUEN, Laboratoire Glyco-MEV EA4358, Normandie Université, Rouen, France; ^2^Unité de Glycobiologie Structurale et Fonctionnelle (UGSF), UMR 8576, CNRS, Université de Lille, Lille, France

**Keywords:** microalgae, post-translational modification, *N*-glycosylation, *O*-glycosylation, protein, biopharmaceuticals, endoplasmic reticulum, Golgi apparatus

## Abstract

The term microalga refers to various unicellular and photosynthetic organisms representing a polyphyletic group. It gathers numerous species, which can be found in cyanobacteria (i.e., *Arthrospira*) as well as in distinct eukaryotic groups, such as Chlorophytes (i.e., *Chlamydomonas* or *Chlorella*) and Heterokonts (i.e., diatoms). This phylogenetic diversity results in an extraordinary variety of metabolic pathways, offering large possibilities for the production of natural compounds like pigments or lipids that can explain the ever-growing interest of industrials for these organisms since the middle of the last century. More recently, several species have received particular attention as biofactories for the production of recombinant proteins. Indeed, microalgae are easy to grow, safe and cheap making them attractive alternatives as heterologous expression systems. In this last scope of applications, the glycosylation capacity of these organisms must be considered as this post-translational modification of proteins impacts their structural and biological features. Although these mechanisms are well known in various Eukaryotes like mammals, plants or insects, only a few studies have been undertaken for the investigation of the protein glycosylation in microalgae. Recently, significant progresses have been made especially regarding protein *N*-glycosylation, while *O*-glycosylation remain poorly known. This review aims at summarizing the recent data in order to assess the state-of-the art knowledge in glycosylation processing in microalgae.

## Introduction

All microalgae share two common features: they are unicellular and photosynthetic organisms. According to the literature, more than thirty thousand organisms fall into this definition (Guiry, 2012; Rumin et al., 2020). Beside these common features, microalgae species exhibit a broad diversity of morphology, size (ranging from a few to one hundred micrometers), physiology and metabolism. This diversity results from various adaptation strategies allowing them to colonize very different habitats going from freshwaters and oceans to terrestrial environments (Brodie et al., 2017). Microalgae represent a polyphyletic group meaning that they spread in distinct phyla ranging from Cyanobacteria to Eukaryotes (Burki et al., 2020). As far as eukaryotic species are concerned, most of them are distributed in two supergroups: the Archaeplastida and the Chromalveolata lineages arising from series of endosymbiotic events leading to various photosynthetic organisms (Gould et al., 2008). The first endosymbiosis is thought to have arisen between 1 and 1.5 billion years ago. During this event, a cyanobacterium was engulfed by an eukaryotic host cell that gave birth to three photosynthetic lineages: the Chlorophytes, the Rhodophytes and the Glaucophytes, in which cells are characterized by the presence of primary plastids corresponding to the ancestral cyanobacterium. These three photosynthetic lineages form together the Archaeplastida supergroup, also named the “green lineage” (Rodríguez-Ezpeleta et al., 2005). Then, during secondary endosymbiotic events, some of these eukaryotic cells containing a primary plastid were engulfed by another eukaryotic cell, leading to new photosynthetic cells in which photosynthesis occurs in secondary plastids. Organisms that derived from a secondary endosymbiosis involving a rhodophyte as a host cell belongs to the Chromalveolata lineage (Cavalier-Smith, 1999; Keeling, 2009). In addition, a few photosynthetic unicellular organisms are belonging to other Eukaryotic supergroups. For example, the Euglenid group, belonging to the Excavate supergroup, encompasses several freshwater and marine or brackish phototrophic species that are spread out in Euglenales and Eutreptiales, respectively (Vesteg et al., 2019). These phototrophic species are thought to have emerged recently (about 600 million years ago) as the result of a secondary endosymbiosis between an Euglenid host cell and a prasinophyte green alga (Jackson et al., 2018).

Taking advantage of this huge diversity, industrials used microalgae since the 1950’s in various applications ranging from food industry (e.g., pigments extraction; Novoveská et al., 2019) to biofuel production or wastewaters treatments (Oey et al., 2016; Gilmour, 2019; Li et al., 2019). However, the diversity of microalgae metabolisms and the remaining number of unknown species, still represent an untapped potential. This is illustrated through the exponential increase of publications regarding microalgae during the last 10 years: more than 5,700 papers dealing with microalgae have been published in 2018 worldwide representing twice the number of publications in 2010 (Rumin et al., 2020). Furthermore, the advances in genome sequencing technologies allow now access to numerous microalgae genomes (Fu et al., 2019), that facilitate the development of molecular tools for studying metabolic processes in these organisms. Currently, DNA recombinant technology and transgenesis have been successfully implemented in some microalgae. In this context, microalgae have been investigated as emerging industrial platforms for the production of high value-added biopharmaceuticals (Barolo et al., 2020; Dehghani et al., 2020; Rosales-Mendoza et al., 2020). Indeed, microalgae are easy and fast to grow, safe and cheap making them attractive expression systems for the production of therapeutic proteins (Hempel and Maier, 2016; Rosales-Mendoza et al., 2020). Nowadays, the most biological expression systems used for the production of recombinant proteins are bacteria, yeast and mammalian cells (Walsh, 2014). One of the critical issues for the choice of an heterologous system is its capacity of protein glycosylation that is required for the biopharmaceutical biological activity. Apart from efforts to engineer and humanize the *N*-glycosylation pathway in plants, mammalian cells are currently the only system able to synthesize proteins bearing glycan structures close to the human ones, even if differences might subsist. For example, the sialic acids present in the terminal position of CHO *N*-glycan structures are linked in α(2, 3) whereas they are linked in α(2, 6) in human *N*-glycans (Bragonzi et al., 2000). Despite this difference, most of the glycosylated biopharmaceuticals are to date produced in Chinese Hamster Ovary (CHO) cells (O’Flaherty et al., 2017).

The term glycosylation refers to the processes leading to the synthesis of oligosaccharides that are then attached to another molecule like a protein. Glycosylation pathways comprise numerous distinct steps, starting with the cytosolic synthesis of nucleotide-sugars that are used in the Golgi apparatus as donor substrates by specific glycosyltransferases involved in the synthesis of the oligosaccharide moiety. In Eukaryotes, glycoproteins can be distinguished according to the site of glycan attachment on the protein. The attachment of oligosaccharide occurs either on the amide group of an asparagine (Asn) residue (*N*-glycosylation) or on the hydroxyl group of a serine (Ser), a threonine (Thr), or an hydroxyproline residue (*O*-glycosylation). Glycans attached to proteins regulate fundamental biological functions such as cell adhesion, molecular trafficking, control of growth, morphogenesis, adaptation to biotic and abiotic stresses and receptor activation (Schjoldager and Clausen, 2012; Varki, 2017). Moreover, glycosylation of proteins is crucial for their half-life, stability, immunogenicity, secretion and biological activity (Lingg et al., 2012; Van Beers and Bardor, 2012; Zhang et al., 2013).

Although these mechanisms are well described in various Eukaryotes like vertebrates (Moremen et al., 2012; Stanley et al., 2017), plants (Nguema-Ona et al., 2014; Strasser, 2016; Schoberer and Strasser, 2018) or insects (Walski et al., 2017), only few studies have been undertaken to investigate the protein glycosylation pathways in microalgae. Recently, significant progresses have been made regarding especially the *N*-glycosylation in microalgae, while *O*-glycosylation remain poorly known. This review reports on recent findings and summarizes the current knowledge in the *N*- and *O*-glycosylation pathways in microalgae.

## *N*-Glycosylation

### General Features of Eukaryotic *N*-Glycosylation

In Eukaryotes, protein *N*-glycosylation process can be divided in three major steps: the synthesis of the oligosaccharide moiety on a lipid carrier, called the lipid-linked oligosaccharide (LLO), the transfer of this oligosaccharide precursor on the target protein and the maturation of the protein *N*-linked glycans. The two first steps occur in the Endoplasmic Reticulum (ER) while the maturation and further elongation of the protein *N*-glycans take place in the Golgi apparatus.

**FIGURE 1:**
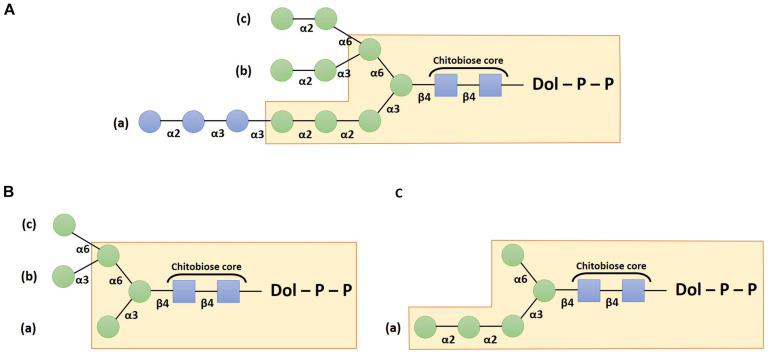
Schemes depicting the structure of the dolichol pyrophosphate oligosaccharide precursor **(A)**, the canonical Man_5_GlcNAc_2_ structure **(B)** and the non-canonical Man_5_GlcNAc_2_ structure **(C)**. Structures are drawn according to the Symbol Nomenclature For Glycans (SNFG) (Neelamegham et al., 2019). Blue squares: *N*-acetylglucosamine residues; green circles: mannose and blue circles: glucose residues.

The assembly of the oligosaccharide moiety requires several enzymes called Asparagine-linked glycosylation (ALG) that act according to well-established sequential steps. It starts on the cytosolic side of the ER with the addition of two *N*-acetylglucosamine (GlcNAc) residues on a dolichol pyrophosphate (PP-Dol) lipid carrier that is embedded in the ER membrane. A first GlcNAc residue is transferred from UDP-GlcNAc to the PP-Dol by the GlcNAc-1-phosphotransferase ALG7 (also called DPAGT1 in mammals) (Bretthauer, 2009) and then the ALG13/ALG14 complex adds the second GlcNAc residue to form GlcNAc_2_-PP-Dol (Bickel et al., 2005; Gao et al., 2005) in which the two GlcNAc linked in β(1,4) correspond to the chitobiose core unit (Figure 1). Thereafter, the oligosaccharide is extended sequentially under the activity of several enzymes which add overall five mannose (Man) residues on the chitobiose to form Man_5_GlcNAc_2_-PP-Dol. This intermediate structure is translocated across the ER membrane by a flip-flop mechanism involving flippases like RFT1 (Frank et al., 2008). Then, the synthesis continues within the ER luminal compartment with the addition of four other Man residues leading to an oligosaccharide lipid precursor Man_9_GlcNAc_2_-PP-Dol thanks to the respective action of ALG3, ALG9, and ALG12. Finally, the glucosyltransferases ALG6, ALG8, and ALG10 transfer three glucose (Glc) residues to build the final structure Glc_3_Man_9_GlcNAc_2_-PP-Dol (Burda and Aebi, 1998; Farid et al., 2011; Bloch et al., 2020; Figure 1A). Afterward, the oligosaccharide moiety is transferred “*en bloc*” from the precursor onto a specific asparagine residue belonging to the *N*-glycosylation consensus site Asn-X-Ser/Thr/Cys (where X cannot be a proline) of a newly synthesized protein (Matsui et al., 2011; Schwarz and Aebi, 2011). The transfer occurs either co- or post-translationally.

Once the oligosaccharide has been transferred on the target protein, the two terminal Glc residues are trimmed by the α-glucosidases I and II. The resulting Glc_1_Man_9_GlcNAc_2_ structure is then involved in the quality control by interacting with calnexin or calreticulin chaperone that contribute to glycoprotein folding (for recent reviews please refer to Strasser, 2018; Adams et al., 2019). Thus, the involvement of this *N*-glycan precursor in the protein quality control cycle justifies that ER processing steps of protein *N*-linked glycans are highly conserved in most of the Eukaryotes. However, exceptions have been described, especially in parasitic species that lack some ER luminal ALG (Samuelson et al., 2005). For example, in *Toxoplasma gondii*, *Cryptosporidium parvum*, and *Tetrahymena pyriformis*, the luminal ALG responsible for the addition of the four last Man residues are absent. Thus, the synthesis of the oligosaccharide precursor stops prematurely leading to a structure harboring only five mannose residues (Yagodnik et al., 1987; Garénaux et al., 2008; Haserick et al., 2017). Moreover, in *Cryptococcus neoformans*, *Trypanosoma brucei*, and *Trypanosoma cruzi*, ALG6, ALG8 and ALG10 are missing, resulting in a non-glucosylated precursor Man_9_GlcNAc_2_-PP-Dol (De La Canal and Parodi, 1987; Parodi, 1993).

Subsequently, correctly folded glycoproteins leave the ER and transit through the Golgi apparatus where α(1,2)-Man residues are first removed (Benyair et al., 2015). This process involves several isoforms of α-mannosidases I and leads to glycoproteins bearing Man_5_GlcNAc_2_ structures (Figure 1B). Whereas these early Golgi steps are common in most Eukaryotes, following maturation steps greatly differ according to the Golgi enzyme repertoire, giving rise to various distinct structures between species (Wang et al., 2017). The synthesis of the complex *N*-glycan structures depends especially on the activity of the β(1,2)-*N*-acetylglucosaminyltransferase I (GnT I), a key enzyme that transfers a GlcNAc residue on the α(1,3)-Man attached to the chitobiose core. In organisms where *N*-glycosylation is GnT I-dependent (i.e., plants, insects or mammals), the attachment of the GlcNAc residue is followed by the removing of two outer terminal Man residues by the α-mannosidase II (Rose, 2012). Then, a β(1,2)-*N*-acetylglucosaminyltransferase II (GnT II) adds another GlcNAc residue on the α(1,6)-Man attached to the *N*-glycan chitobiose core. The resulting GlcNAc_2_Man_3_GlcNAc_2_ structures can be further “decorated” by diverse glycosyltransferases such as fucosyltransferases, xylosyltransferases, galactosyltransferases, or sialyltransferases. In mammals, supplemental *N*-acetylglucosaminyltransferases add a third, a fourth and sometimes a fifth GlcNAc leading to the formation of tri- and tetra-antennary *N*-glycan structures (Wang et al., 2017).

### *N*-Glycosylation Pathways in Microalgae

In microalgae, current knowledge regarding the *N*-glycosylation processes is available essentially in species belonging to the Chlorophytes (Table 1). Among these, the Chlorophyceae *Chlamydomonas reinhardtii* and the Trebouxiophycea *Chlorella vulgaris* have been the most investigated microalgae so far (Mathieu-Rivet et al., 2013; Vanier et al., 2017; Lucas et al., 2018, 2020; Schulze et al., 2018; Mócsai et al., 2019, 2020a, b; Oltmanns et al., 2020). Concerning microalgae with secondary plastids, the structural data available regarding *N*-glycans have been obtained from the diatom *Phaeodactylum tricornutum* (Baïet et al., 2011; Vanier et al., 2015; Lucas et al., 2018) and from the Euglenoid *Euglena gracilis* (De La Canal and Parodi, 1985; O’Neill et al., 2017).

**TABLE 1:** Table summarizing published results regarding the *N*- and *O*-glycosylation processes in microalgae species.

Microalgae species	References	New insights about
		***N-glycosylation***

**Chlorophyta**		
*Chlorella* sp.	Mócsai et al., 2019	First insights regarding *N*-glycosylation pathway in *Chlorella vulgaris*. Identification of *O*-methylated oligomannoside *N*-glycans.
	Mócsai et al., 2020a, b	Analysis of *N*-glycans in a strain collection and commercial products derived from *C. vulgaris* and *C. sorokiniana* highlighted for the first time a huge heterogeneity of *N*-glycan structures and the presence of arabinose residues in *N*-glycans that has never been reported before.
*Chlamydomonas reinhardtii*	Mathieu-Rivet et al., 2013	First glycomic and glycoproteomic analysis carried out on total and membrane-bound proteins demonstrated that the *N*-glycosylation results from a GnT I-independent process.
	Vanier et al., 2017	Revision of the ER *N*-glycosylation pathway based on the demonstration that *C. reinhardtii* synthesizes a non-canonical Man_5_GNAc_2_. Heterologous expression of functional GnT I was shown to have no impact on *C. reinhardtii N*-glycosylation process, although physiology of the transformant cells was impaired.
	Lucas et al., 2018	First Structural analysis of the LLO confirming the absence of luminal ER mannosylation steps.
	Schulze et al., 2018	First functional analysis of the xylosyltransferase XylTA. The regulation of *N*-glycans trimming by ManIA isoform depends on the presence of the core β(1,2)-xylose residue.
	Lucas et al., 2020	Analysis of the respective roles of the two xylosyltransferases, XylTA and XylTB, in the maturation of the *N*-glycans. Identification of additional XylT putative candidates.
	Oltmanns et al., 2020	The knockdown of a fucosyltransferase candidate did not impair the fucosylation of the *N*-glycans.
*Botryococcus braunii*	Schulze et al., 2017	A *N*-glycoproteomic analysis performed on total cell extract revealed *N*-glycan structures synthesized *via* a GnT I-dependent pathway. Mature *N*-glycans harbor *O*-methylated hexoses.
*Scherffelia dubia*	Grunow et al., 1993	Analysis of proteins from Golgi membrane fractions by Eastern blot suggests the presence of complex and oligomannoside *N*-glycans.
*Tetraselmis striata*	Gödel et al., 2000	Glycosidase treatments of flagellar proteins, followed by Eastern blot analysis showed the presence of complex and oligomannoside *N*-glycans.
*Volvox carteri*	Müller et al., 1984	First evidence of a truncated LLO synthesis pathway in a microalgae specie.
	Balshüsemann and Jaenicke, 1990	Enzyme sequencing experiments suggested that secreted proteins carry complex *N*-glycans bearing a core β(1,2)-xylose residue.
**Rhodophyta**		
*Porphyridium* sp.	Levy-Ontman et al., 2011	*N*-glycans isolated from a cell wall glycoprotein are *O*-methylated oligomannosides carrying xylose residues.
	Levy-Ontman et al., 2014	Identification of genes encoding enzymes related to the ER *N*-glycosylation pathway.
	Levy-Ontman et al., 2015	Identification and functional characterization of the α(1,3)-Glucosidase II acting within the ER.
**Diatoms**		
*Phaeodactylum tricornutum*	Baïet et al., 2011	First report of the *N*-glycan structures in the diatom *P. tricornutum* Functional characterization of GnT I, which was the first glycosyltransferase from microalgae to have been characterized.
	Vanier et al., 2015	Characterization of the *N*-glycans harbored by a recombinant monoclonal antibody directed against the Hepatitis B virus surface antigen produced in *P*. *tricornutum.*
	Lucas et al., 2018	Elucidation of the LLO structure.
	Zhang et al., 2019	Functional characterization of a core fucosyltransferase (FucT) and demonstration of the existence of a sub-compartmentation of Golgi enzymes (GnT I and FucT) as reported in plants and mammals.
**Euglenozoa**		
*Euglena gracilis*	De La Canal and Parodi, 1985	First evidence of the synthesis of the Glc_3_Man_9_GlcNAc_2_-PP-Dol precursor.
	O’Neill et al., 2017	Mass spectrometry analysis carried out on PNGase F released *N*-glycans showed that the major part of the *N*-glycans are oligomannosides. Minor part of the *N*-glycan population possesses a non-reducing extremity modified by the addition of a 2-aminoethylphosphonate group.

		***O-glycosylation***

**Chlorophyta**		
*Chlorella vulgaris*	Lamport and Miller, 1971	First report of hydroxyproline-linked arabinosides in the cell wall.
*Chlamydomonas reinhardtii*	Miller et al., 1972	The alkaline hydrolysis of a crude cell wall fraction released a striking variety of hydroxyproline-*O*-glycosides, including mostly hydroxyproline-linked arabinosides with one or two Ara residues, as well as hydroxyproline-*O*-galactose.
	Bollig et al., 2007	First elucidation of the *O*-glycosylation pathway in *C. reinhardtii. O*-glycoprotein from chaotrope-soluble cell wall shows extensins like structure with a core Hyp-*O*-Ara-Ara. Two arabinosyltransferases, one galactofuranosyltransferase and methyltransferases might be involved in the *O*-glycan biosynthesis.
	Keskiaho et al., 2007	Characterization of the prolyl-4-hydroxylase, which efficiently hydroxylates the proline residues of synthetic peptides. It’s down-regulation in *C. reinhardtii* affect the assembly of its cell wall.
	Saito et al., 2014	Characterization of the peptidyl-serine α-galactosyltransferase (SERGT1) from the GT96 CAZy family. This enzyme is responsible for the transfer of a single α-galactopyranose residue to each Ser residue in Ser-(Hyp)_4_.
*Volvox carteri*	Balshüsemann and Jaenicke, 1990	Enzyme sequencing experiments suggest that *O*-glycans are exclusively bound to threonine residues and correspond to short oligosaccharides (up to three sugar residues) composed of Ara, Gal and Xyl.
*Scenedesmus obliquus*	Voigt et al., 2014	Cell wall of *S. obliquus* contains a glycoprotein homolog to the *C. reinhardtii* cell wall GP3B.
**Charophyta**		
*Micrasterias denticulata*	Eder et al., 2008	Biochemical analyses of cell wall constituents revealed various plant-like AGPs epitopes.
**Euglenozoa**		
*Euglena gracilis*	O’Neill et al., 2017	No *O*-glycan specific signature has been observed in *E. gracilis* after PNGase F and β-elimination treatments.
		***Nucleotide-sugars and transporters***
*Chlamydomonas reinhardtii*	Mathieu-Rivet et al., 2017	Bioinformatic analysis of nucleotide-sugar transporters.
*Phaeodactylum tricornutum*	Zhang et al., 2019	Functional characterization of a GDP-fucose transporter, first nucleotide-sugar transporter from microalgae to have been characterized.

#### Synthesis of the Precursor in the ER

In *C. reinhardtii*, the synthesis of the LLO in the ER stops prematurely as this organism lacks the luminal mannosyltransferases ALG9 and ALG12, as well as the glucosyltransferase ALG10 (Mathieu-Rivet et al., 2013; Vanier et al., 2017). In addition, a LLO-released oligosaccharide Glc_3_Man_5_GlcNAc_2_ moiety has been identified by multistage tandem mass spectrometry (Lucas et al., 2018). This oligosaccharide is transferred on proteins *via* the oligosaccharyltranferase (OST) complex for which seven homolog subunits have been predicted based on the genome analysis (Mathieu-Rivet et al., 2013). Then, glycosylated proteins are submitted to the control quality cycle that involves α-glucosidases I and II as well as the calnexin and calreticulin chaperones. As a consequence, glycoproteins that exit the ER harbor a non-canonical Man_5_GlcNAc_2_ *N*-glycan exhibiting a linear trimannosyl sequence linked to the β-Man residue (Figure 1C) instead of the canonical Man_5_GlcNAc_2_ (Figure 1B). A first study carried out previously in the colonial microalgae *Volvox carteri*, which is phylogenetically closely related to *C. reinhardtii*, also highlighted the absence of ER luminal mannosylation steps (Müller et al., 1984). Thus, regarding these features, ER steps in both *C. reinhardtii* and *V. carteri* appear to be similar to those described in *T. gondii* (Garénaux et al., 2008), *C. parvum* (Haserick et al., 2017) and *T. pyriformis* (Yagodnik et al., 1987; Table 1).

In contrast, data reported in other microalgae species suggest that the oligosaccharide precursor is synthesized according to a more conventional process. Thus, ER pathways in *C. vulgaris* and *Botryococcus braunii* appears to be similar to those described in plants since these microalgae synthesizes oligomannosides ranging from Man_5_GlcNAc_2_ to Man_9_GlcNAc_2_ (Schulze et al., 2017; Mócsai et al., 2019). In addition, the structural analysis of a cell wall glycoprotein from the red microalgae *Porphyridium* sp. has revealed the presence of *N*-glycans containing eight to nine Man residues (Levy-Ontman et al., 2011). These results are consistent with the bioinformatic prediction of genes encoding for ER enzymes (Levy-Ontman et al., 2014). In *E. gracilis*, labeling assays of protein-linked oligosaccharides have demonstrated that Glc_3_Man_9_GlcNAc_2_-PP-Dol is synthesized before transfer of the carbohydrate moiety on proteins (De La Canal and Parodi, 1985). More recently, O’Neill et al. (2017) have shown using mass spectrometry that major *N*-glycans in this specie correspond to oligomannoside structures. Proteins from *P. tricornutum* also carry oligomannoside *N*-glycans having five to nine Man residues (Baïet et al., 2011). In agreement with this *N*-glycan profile, *P. tricornutum* LLO oligosaccharide has been identified as being Glc_2_Man_9_GlcNAc_2_ that is missing the terminal α(1,2)Glc residue (Lucas et al., 2018).

#### GnT I: To Have or Not to Have

In most Eukaryotes, the Golgi maturation steps depends on the transfer by GnT I of a GlcNAc residue on the arm (a) of the canonical Man_5_GlcNAc_2_ (Figure 1B), thus opening the door to the formation of complex-type *N*-glycans. This key step does not seem to be a general rule on the microalgae that have been studied so far. In *P. tricornutum*, although no structure harboring terminal GlcNAc residues has been detected in PNGase-released *N*-glycans, a genomic sequence encoding for a GnT I has been shown to efficiently restore the CHO *Lec1* cell line that is deficient for this enzyme activity. This result demonstrated that the paucimannosidic fucosylated structures Man_3_FucGlcNAc_2_ identified in the protein *N*-glycan profiles of *P. tricornutum* likely results from a GnT I-dependent process (Baïet et al., 2011). In contrast, glycoproteins from *C. reinhardtii* are processed through a GnT I-independent pathway (Mathieu-Rivet et al., 2013; Vanier et al., 2017). No gene candidate has been identified in the genome by search for sequence homology using functional GnT I from others species as queries. Furthermore, the heterologous expression of GnT I from *Arabidopsis thaliana* or *P. tricornutum* did not impact the *N*-glycan profile of *C. reinhardtii* proteins. This is consistent with the fact that this green microalga synthesized a non-canonical Man_5_GlcNAc_2_ (Figure 1C) that is not an acceptor substrate for GnT I (Vanier et al., 2017). Thus, glycoproteins harboring the non-canonical Man_5_GlcNAc_2_ are submitted in the Golgi apparatus to the action of glycosyltransferases responsible for the addition of decorations. Mass spectrometry analyses carried out on *C. reinhardtii* secreted and membrane-bound proteins have shown that mature *N*-glycans are partially *O*-methylated Man_3_GlcNAc_2_ to Man_5_GlcNAc_2_ substituted by one or two Xyl residues (Mathieu-Rivet et al., 2013), and for a minor part by one fucose residue (Oltmanns et al., 2020). Recently, Schulze et al. (2018) and Lucas et al. (2020) have showed that the first Xyl is linked in β(1,2) to the β-Man *via* the action of the xylosyltransferase A (XylTA) similarly to the plant xylosylation process. In contrast, the xylosyltransferase B (XylTB) is responsible for the transfer of a second residue on the linear trimannosyl branch of the Man_5_GlcNAc_2_ structure. However, although it is clearly established that these two XylT play a major role in the *N*-glycan xylosylation processing, the remaining presence of structures containing Xyl residues in a double knockdown mutant *XylTA* × *XylTB* has suggested that other uncharacterized enzymes could also contribute to the *N*-glycan xylosylation in *C. reinhardtii*. The fucosylation mechanism remains uncleared as the analysis of an insertional mutant in which the candidate gene encoding for a putative FucT was disrupted, did not affect *N*-glycans harboring Fuc residues (Oltmanns et al., 2020). In addition, a bioinformatic analysis of other microalgae genomes showed that other Chlorophyta species like *Ostreococcus lucimarinus*, *Ostreococcus tauri*, or *V. carteri* would lack GnT I enzymatic activity (Mathieu-Rivet et al., 2014), which suggest that the GnT I-independent process described in *C. reinhardtii* would not be an exception. However, recent structural data obtained in other species indicate that the absence of GnT I is not a common feature in Chlorophyta (Table 1). Indeed, in *B. braunii*, traces amount of Man_5_GlcNAc_2_ bearing a terminal GlcNAc at the non-reducing end has been detected, in addition to the presence of a genomic sequence sharing a strong homology with *A. thaliana* GnT I (Schulze et al., 2017). In *C. vulgaris*, an *in vitro* GlcNAc-transferase assay on *N*-glycans showed that Man_5_GlcNAc_2_ was converted into GlcNAcMan_5_GlcNAc_2_ (Mócsai et al., 2019). Moreover, it was shown that the GlcNAcMan_5_GlcNAc_2_ synthesized by *C. vulgaris* was substrate for core 6-fucosyltransferase, which depends on the presence of terminal GlcNAc (Mócsai et al., 2020b). Altogether, this favors the existence of a GnT I-dependent processing of the *N*-glycans in the Golgi apparatus of these species. In addition, the study of two strain collections from *C. vulgaris* and *Chlorella sorokiniana* also revealed heterogeneous *N*-glycan structures with both arabinose and galactose occurring as furanose as well as pyranose forms (Mócsai et al., 2020a, b), that constitute an unprecedented discovery among the Eukaryotes.

## *O*-Glycosylation

### General Features of Eukaryotic *O*-Glycosylation

As for *N*-glycosylation, several families of enzymes orchestrate *O*-glycosylation pathways. Unlike *N*-glycosylation in which the first ER steps are conserved in most Eukaryotes, the *O*-glycosylation of proteins encompasses various distinct processes. Some of them start in the ER and continue in the Golgi apparatus, while others occur exclusively in the Golgi apparatus.

*O*-glycosylation involves an oxygen-carbon bond between the hydroxyl group of a Ser or a Thr residue of the protein and the oligosaccharide chain in mammals (Bennett et al., 2012) while in plants, *O*-glycosylation occurs essentially in hydroxyproline residue (Hyp; Nguema-Ona et al., 2014; Seifert, 2020). In most eukaryotes including humans, *O*-glycans do not present a common structure or a consensus sequence. For example, *O*-glycans in yeasts are composed of multiple Man residues attached to a Ser or a Thr (Schoberer and Strasser, 2018; Barolo et al., 2020). In mammals, most of *O*-glycans were found on mucins. Mucins represent large glycoproteins with three domains: (i) a cytoplasmic tail; (ii) a single transmembrane spanning region and (iii) an extracellular domain. The extracellular domain contains a repeating peptide motif with numerous proline (Pro), Ser and Thr residues. The first monosaccharide attached to the mucin is usually β-GalNAc but can also be β-GlcNAc, α-GalNAc, α-Man or other monosaccharides (Bennett et al., 2012; Schoberer and Strasser, 2018). More than 20 different UDP-GalNAc polypeptide *N*-acetylgalactosaminyltransferases can be involved in the GalNAc attachment. This GalNAc is further modified by the stepwise attachment of different monosaccharides such as galactose (Gal), GlcNAc, sialic acid and fucose giving rise to diverse mucin-type core *O*-glycans that play crucial roles in many biological processes (Schjoldager and Clausen, 2012; Mewono et al., 2015).

In plants, the main *O*-glycosylated proteins belong to a large group of glycoproteins known as Hydroxyproline-rich-glycoproteins (HRGPs). HRGPs are involved in many aspects of plant growth and development. They consist in a superfamily of plant cell wall proteins that are divided into three major multigene families: the highly glycosylated arabinogalactan proteins (AGPs), the moderately glycosylated extensins (EXTs) and the low glycosylated proline-rich proteins. The *O*-glycosylation of HRGPs results from two consecutive post-translational modifications involving the hydroxylation of Pro (Hyp) residues by prolyl 4-hydroxylases in the ER and the subsequent *O*-glycosylation in the Golgi apparatus of some, but not all, Hyp residues by glycosyltransferases before being transported to their final location within or outside the cell (Nguema-Ona et al., 2014; Seifert, 2020). Overall, *O*-glycan cores in plants present a Gal residue attached to a Ser or an unique arabinose (Ara) residue attached to an Hyp. The monosaccharide being incorporated and the level of glycosylation depends on the glycoproteins families (AGPs, EXTs or proline-rich proteins). The *O*-glycans of AGPs are composed of short oligoarabinoside chains containing up to four residues and of a larger β(1,3)-linked galactan backbone with β(1,6)-linked side chains containing galactose, arabinose and, sometime fucose, rhamnose, or glucuronic acid. The structure of arabinogalactan chains varies between plant species (Nguema-Ona et al., 2014). EXTs contain several Ser-(Hyp)_4_ repeats usually *O*-glycosylated with oligosaccharide chains of up to five arabinose units on each Hyp (Velasquez et al., 2011, 2015; Ogawa-Ohnishi et al., 2013) and a unique galactose on the Ser residue (Saito et al., 2014). *O*-glycosylated Ser-(Hyp)_4_ repeat sequences have also been identified in several other EXT-like chimeras and hybrid EXT glycoproteins, such as arabinogalactan protein-EXTs, Pro-rich protein-EXTs, Leu-rich repeat-EXTs, Pro-rich kinases and formins with an extracellular EXT domain (Velasquez et al., 2015). Moreover, Hyp-*O*-arabinosylation also occurs in single Hyp units in small secreted glycopeptide hormones with up to three arabinose units (Ohyama et al., 2009; Shinohara and Matsubayashi, 2010).

Concerning the enzyme machinery involved in the HRGP synthesis, three groups of arabinosyltransferases (AraTs) have been identified: hydroxyproline *O*-arabinosyltransferase 1 (HPAT1 to HPAT3), reduced residual arabinose (RRA1 to RRA3) and xyloglucanase 113 (XEG113). These transferases have been demonstrated to be responsible for the sequential addition of the innermost three arabinose residues (Egelund et al., 2007; Ogawa-Ohnishi et al., 2013).

### *O*-Glycosylation in Microalgae

Very few articles related to the *O*-glycosylation pathways in microalgae are available to date. So far, *C. reinhardtii* is the main microalga that has been investigated regarding protein *O*-glycosylation.

#### Extensin-Like *O*-Glycoproteins

Bollig et al. (2007) have investigated the structure of linear glycans *O*-linked to Hyp residues of *C. reinhardtii* proteins, showing some similarities with plant *O*-glycans. Indeed, they identified a *O*-glycan core Hyp-*O*-Ara-Ara, which is consistent with previous results reported by Miller and coworkers (Miller et al., 1972). This suggests a certain level of conservation of the extensin structures within the green lineage.

**FIGURE 2:**
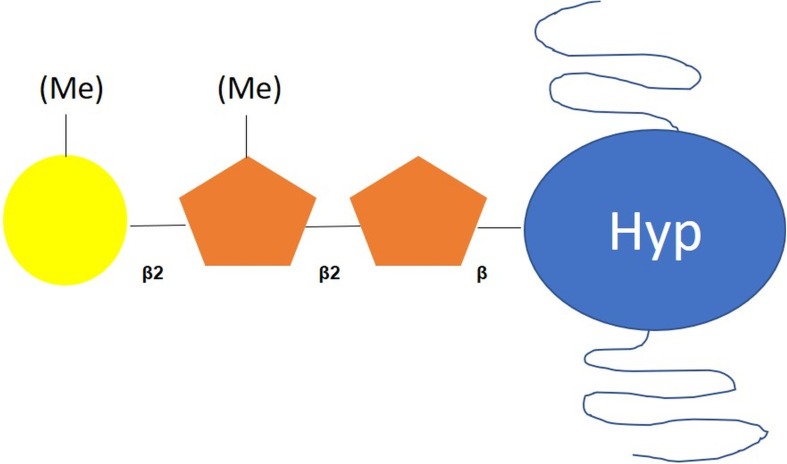
Scheme of *O*-glycan motif harbored by proteins in *C. reinhardtii*. According to Bollig et al. (2007), *O*-glycans attached to *C. reinhardtii* proteins are composed of a Hyp-O-Ara-Ara core substituted with methylated galactofuranose residues. Hyp: hydroxyproline; orange pentagon: arabinofuranose; yellow circle: galactofuranose, Me: methyl group.

*O*-glycosylated Hyp residues have been identified in chaotrope-soluble glycoproteins, which constitute the vegetative outer cell wall in *C. reinhardtii* (Bollig et al., 2007). Mass spectrometry and NMR analyses have indicated the presence of mainly Ara and Gal, followed by Glc, Xyl and Man residues (Bollig et al., 2007). Bollig et al. (2007) have demonstrated that glycans *O*-linked to Hyp residue are composed of a β(1-2)-linked L-Ara disaccharide substituted with galactofuranose (Gal*f*) residues and *O*-methylation, two modifications not reported in plants (Bollig et al., 2007; Mathieu-Rivet et al., 2017; Barolo et al., 2020; Figure 2). Little information is available concerning the enzymes involved in this process in *C. reinhardtii*. However, a prolyl-4-hydroxylase has been characterized (Keskiaho et al., 2007). This enzyme efficiently hydroxylates the Pro residues of synthetic peptides and its down-regulation affect the assembly of a proper cell wall, which is consistent with the role of hydroxyproline residues in the attachment of the oligosaccharide moiety (Keskiaho et al., 2007).

In addition, few papers have reported data regarding the composition of cell wall glycoproteins of others microalgae. Hyp-linked arabinosides have been reported in the green alga *C. vulgaris* (Lamport and Miller, 1971). Balshüsemann and Jaenicke (1990) have described *O*-linked oligosaccharides in the glycoprotein pheromones of *V. carteri*. These short oligosaccharide chains (up to three residues) are composed of Ara, Gal and Xyl bound to Thr residues (Balshüsemann and Jaenicke, 1990). Eder et al. (2008) have detected in the cell wall of the green alga *Micrasterias denticulata* various plant-like AGPs epitopes by combination of cell imaging and biochemical approaches. Cell-wall glycoprotein number 1, 2, and 3 (GP1, GP2, and GP3, respectively) are hydroxyproline-rich glycoproteins that co-polymerize to form the W6 layer of *C. reinhardtii* cell wall. W6 layer is one of the three major outer layers of the *C. reinhardtii* cell wall that can be solubilized from living cells with chaotropes (Monk, 1988). Voigt and collaborators have studied the ultrastructure of cell wall glycoproteins of *V. carteri* and the green alga *Scenedesmus obliquus*. A multi-layered cell wall similar to the GP3 of *C. reinhardtii* has been reported although its proportion in Hyp is considerably lower (Voigt et al., 2014). Glycoproteins similar to GP1 have also been found in other *Chlamydomonas* species (*eugametos* and *incerta*) (Goodenough et al., 1986) but are absent in *Volvovaceae Gonium pectoral* and *V. carteri* (Voigt et al., 2014). The chaotrope-soluble cell wall glycoprotein GP1 is the only polypeptide with an even higher proportion of Hyp (35%) occurring in vegetative *C. reinhardtii* cells (Voigt et al., 2009). In contrast, GP2 and GP3 have been found in all studied *Volvovaceae* species. Putative homologs of GP3 have also been detected on the cell walls of some *Zygnematales* using a polyclonal antibody raised against the glycosylated GP3B isoform of *C. reinhardtii* (Voigt et al., 2014).

#### Arabinosylation of *O*-Glycans in Microalgae

Bollig et al. (2007) have proposed that two arabinosyltransferases are responsible for the addition of the first two Ara residues onto Hyp followed by the action of a galactofuranosyltransferase (Gal*f*T) in *C. reinhardtii* (Figure 2 and Table 1). These two arabinoses are arabinofuranosyl (Ara*f)* rather than arabinopyranosyl (Ara*p*) residues. UDP-L-Ara*p* is first synthesized in the cytosol from UDP-Xyl and is then converted into UDP-L-Ara*f* through the action of a specific mutase (Bollig et al., 2007). An UDP-L-Ara*p* mutase sharing 78% of identity with AtRGP1 that catalyzes the conversion of UDP-L-Ara*p* into UDP-L-Ara*f* in *A. thaliana* (Rautengarten et al., 2011) has been purified from the cytosol of *C. reinhardtii*. UDP-L-Ara*p* mutase activity has also been detected in microsomal fraction of *C. reinhardtii* (Kotani et al., 2013).

Three putative arabinosyltransferases have been predicted in *C. reinhardtii*’s genome. The Hyp *O*-arabinosyltransferase HPAT, belonging to the CAZy GT95 family, performs the transfer of a β-linked L-Ara to Hyp. Genes encoding homologous transferases have also been found in the genomes of *V. carteri* (Ogawa-Ohnishi et al., 2013), *B. braunii* and *C. vulgaris* (Barolo et al., 2020). The second arabinosyltransferase, RRA (CAZy GT77), transfers L-Ara residues linked in β(1-2) to the Hyp-linked Ara (Velasquez et al., 2015). Based on the recent *in silico* analysis reported by Barolo et al. (2020), this putative enzyme is predicted in the genomes of *Porphyridium purpureum* and *C. vulgaris*. The third one is XEG113, a xyloglucanase that acts as an arabinosyltransferase. XEG113 homolog sequences have been identified in *O. lucimarinus* and *O. tauri*, suggesting the synthesis of closely related extensins in these microalgae (Roycewicz and Malamy, 2014).

#### Galactosylation of *O*-Glycans in Microalgae

UDP-Gal is synthesized from UDP-Glc *via* the epimerization of the C4 hydroxyl group. Whereas several isoforms of UDP-Gal-4-epimerase (UGE) have been found in plants, only one single sequence encoding for a putative GME has been identified in *C. reinhardtii* (Cre04.g214502, CrGME; Rösti et al., 2007). Moreover, it has been shown that the Gal residues present in *C. reinhardtii O*-glycans exhibits the unusual furanose conformation (Gal*f*) (Bollig et al., 2007). Bollig et al. (2007) have proposed that the UDP-Gal*f* residues result from the activity of an UDP-galactopyranose mutase (UGM), which is able to convert UDP-galactopyranose (UDP-Gal*p*) into UDP-Gal*f*. UGMs were found in prokaryotes and a few eukaryotes such as *C. neoformans* or *T. cruzi*. One gene sequence encoding for a putative UGM is predicted in the genome of *C. reinhardtii* (Cre06.g272900). This putative *C. reinhardtii* UGM shares 60% of identity with the UGM from *C. neoformans* (Beverley et al., 2005). In addition, a gene sequence encoding for a putative Gal*f*T (Cre02.g108200) is predicted in *C. reinhardtii* genome (Hung et al., 2016). The presence of Gal*f* has also been reported in the glycosylated toxin “prymnesium” extracted from a red tide microalga *Prymnesium parvum* (Binzer et al., 2019). Genes encoding putative UGM are predicted in JGI phytozome 13 in other microalgae, such as *V. carteri*, *B. braunii* (Chlorococcales), *Coccomyxa subellipsoidea* (Chlorophytes), *Chromochloris zofingiensis*, and *Dunaliella salina*, suggesting the presence of Gal*f* residues on the glycans of these microalgae (unpublished data, personal communication).

One the other hand, a peptidyl-serine α-galactosyltransferase, named SERGT1, has been characterized in *C. reinhardtii* by Saito et al. (2014). This enzyme has been purified from an endosomal fraction and its galactosyltransferase activity has been confirmed by an *in vitro* assay. These results revealed that SERGT1 transfer the single α-galactopyranose residue to Ser residues in Ser-(Hyp)_4_ motifs of EXT, suggesting that *O*-glycosylation of Ser residues can occur in *C. reinhardtii*.

#### Other *O*-Glycosylation Types in Microalgae

Other O-glycosylation types might exist in microalgae. Recently, using computational analysis of available microalgae genomes, Barolo and collaborators have searched for putative candidates involved in protein *O*-glycosylation by comparison with genes encoding enzymes of *O*-glycan pathways in both humans (*Homo sapiens*) and plants (*A. thaliana*) (Barolo et al., 2020). Through this work, it was shown that *C. reinhardtii* exhibits an enzyme repertoire that possess a putative *O*-fucosyltransferase (POFUT 1) that could be involved in protein *O*-fucosylation. This enzyme is also predicted in other microalgae such as *P. tricornutum*, *P. purpureum*, *Nannochloropsis gaditana*, *B. braunii*, and *C. vulgaris* (Barolo et al., 2020). These authors have also highlighted the presence of two putative *O*-mannosyltransferase 1 and 2 (POMT1 and POMT2) activities in *P. purpureum* genome suggesting that *O*-mannosylation of proteins occurs in this microalga as reported in humans and in the yeast, *Saccharomyces cerevisiae* (Barolo et al., 2020). In addition, a putative xylosyltransferase 1 (XylT 1) was found in the genomes of *P. tricornutum* and *N. gaditana*, although the enzymatic activity was not confirmed experimentally. This suggests a possible *O*-xylosylation in these two microalgae (Barolo et al., 2020).

## Methylation of Glycans in Microalgae

Methylation of glycans has been found in the animal kingdom only in worms and mollusks, whereas it is more frequently present in some species of bacteria, fungi and algae (Staudacher, 2012). Methylation has been reported in both *N*- and *O*-glycans in microalgae. Indeed, *O*-methylation of *N*-glycans appears to be a common feature in microalgae species from Archaeplastidae, although it has never been reported in plants. Indeed, Mócsai et al. (2019) have shown that oligomannosidic structures are *O*-methylated in *C. vulgaris* (Mócsai et al., 2019). In addition, *O*-methylated *N*-glycans have also been detected in *B. braunii* (Schulze et al., 2017) and *C. reinhardtii* (Mathieu-Rivet et al., 2013; Vanier et al., 2017; Lucas et al., 2020). Moreover, the biochemical analysis of the 66 kDa cell wall glycoprotein of the Rhodophyta *Porphyridium* sp. revealed the presence of methylated *N*-glycans (Levy-Ontman et al., 2011).

As far as *O*-glycans in microalgae are concerned, Bollig et al. (2007) proposed that two methytransferases specific to *C. reinhardtii* perform methylation of some Gal and Ara residues, which corresponds to the final modification of the protein *O*-glycans in this organism. To the best of our knowledge, none of the enzymes involved in the methylation process has been characterized and the role of the methylation in both *N*- and *O*-glycans remains unknown. In this context, authors have suggested that methylation can confer a protective role to the mature glycans. For example, Wohlschlager et al. (2014) have suggested that *O*-methylated glycans constitute a conserved epitope for the fungal and animal innate immune system. As glycans carrying this modification are present in bacteria, worms, and mollusks, this epitope represents a hitherto unknown target that is recognized by the immune system. Recently, Mócsai et al. (2019) also highlighted that *O*-methylated *N*-glycans are possibly immunogenic. Therefore, this has to be taken into account if pharmaceutical glycoproteins are produced using chlorophytes such as *C. vulgaris* as a cell biofactory. To solve this issue, the authors proposed to identify the *O*-methyltransferase acting on terminal mannose residues and to knockout this enzyme in the future (Mócsai et al., 2019). Such knockout lines could also be used to answer the scientific question of the biological purpose of *N*-glycan methylation.

## Conclusion and Perspectives

To date, little information is available regarding the *N*- and *O*-glycosylation pathways and their regulation in microalgae. Even if the knowledge regarding these protein post-translational modifications has been extended recently, significant efforts remain to be done to characterize these processes in microalgae, especially in the context of using microalgae as cell biofactories where *N*- and *O*-glycosylation pathways remain essential for the biological activities and stability of recombinant proteins (Lingg et al., 2012; Zhang et al., 2013).

In the context of using microalgae as a biopharmaceutical platform for the production of recombinant proteins dedicated to therapeutic applications in humans, it will be crucial to unravel the protein glycosylation pathways and then optimize them in order to mimic human-type *N*- and *O*-glycans through metabolic engineering (Dumontier et al., 2018; Barolo et al., 2020; Rosales-Mendoza et al., 2020). This represents an important challenge for the next decades. However, it would benefit from the recent development of genome-editing tools in microalgae (Daboussi et al., 2014; Mussgnug, 2015; Baek et al., 2016; Nymark et al., 2016; Shin et al., 2016; Wang et al., 2016; Huang and Daboussi, 2017; Dumontier et al., 2018; Slattery et al., 2018; Guzmán-Zapata et al., 2019; Fabris et al., 2020; Moosburner et al., 2020; Park et al., 2020). Moreover, several recent studies carried out in plants have highlighted the feasibility of *N*- and *O*-glycosylation metabolic engineering for the production of humanized recombinant *N*- and *O*-glycoproteins in transgenic plants including for example the production of recombinant IgA1 with defined human-type *N*- and *O*-linked glycans (Bakker et al., 2001; Paccalet et al., 2007; Vézina et al., 2009; Castilho et al., 2010; Castilho and Steinkellner, 2012; Yang et al., 2012; Dicker et al., 2016; Kallolimath et al., 2016).

## Author Contributions

NM-B, EM-R, and MB conceptualized and wrote the manuscript. M-LW-B and PL corrected the manuscript. MB coordinated the work. All authors have read and agreed on the manuscript prior to its submission.

## Conflict of Interest

The authors declare that the research was conducted in the absence of any commercial or financial relationships that could be construed as a potential conflict of interest. The reviewer FA declared a past co-authorship with one of the authors MB to the handling editor.
